# Adipose Derived Stem Cells for Corneal Wound Healing after Laser Induced Corneal Lesions in Mice

**DOI:** 10.3390/jcm6120115

**Published:** 2017-12-05

**Authors:** Marco Zeppieri, Maria Letizia Salvetat, Antonio Beltrami, Daniela Cesselli, Rossella Russo, Ignacio Alcalde, Jesús Merayo-Lloves, Paolo Brusini, Pier Camillo Parodi

**Affiliations:** 1 Department of Ophthalmology, Azienda Ospedaliero Universitaria Santa Maria della Misericordia, Udine 33100, Italy; mlsalvetat@hotmail.it (M.L.S.); brusini@libero.it (P.B.); 2 Department of Pathology, University of Udine, Azienda Ospedaliero Universitaria Santa Maria della Misericordia, Udine 33100, Italy; antonio.beltrami@uniud.it (A.B.); daniela.cesselli@uniud.it (D.C.); 3 Department of Pharmacobiology, University of Calabria, Cosenza 87036, Italy; russors@hotmail.com; 4 Instituto Universitario Fernández-Vega, Fundación de Investigación Oftalmológica, University of Oviedo, Oviedo 33006, Spain; nacho.alcalde@fio.as (I.A.); merayo@fio.as (J.M.-L.); 5 Department of Plastic Surgery, University of Udine, Azienda Ospedaliero Universitaria Santa Maria della Misericordia, Udine 33100, Italy; pcparodi@uniud.it

**Keywords:** adipose derived stem cells, corneal wound healing, stem cell therapy, lipoaspirate, corneal re-epithelization

## Abstract

The aim of our study was to assess the clinical effectiveness of topical adipose derived stem cell (ADSC) treatment in laser induced corneal wounds in mice by comparing epithelial repair, inflammation, and histological analysis between treatment arms. Corneal lesions were performed on both eyes of 40 mice by laser induced photorefractive keratectomy. All eyes were treated with topical azythromycin bid for three days. Mice were divided in three treatment groups (*n* = 20), which included: control, stem cells and basic serum; which received topical treatment three times daily for five consecutive days. Biomicroscope assessments and digital imaging were performed by two masked graders at 30, 54, 78, 100, and 172 h to analyze extent of fluorescein positive epithelial defect, corneal inflammation, etc. Immunohistochemical techniques were used in fixed eyes to assess corneal repair markers Ki67, α Smooth Muscle Actin (α-SMA) and E-Cadherin. The fluorescein positive corneal lesion areas were significantly smaller in the stem cells group on days 1 (*p* < 0.05), 2 (*p* < 0.02) and 3. The stem cell treated group had slightly better and faster re-epithelization than the serum treated group in the initial phases. Comparative histological data showed signs of earlier and better corneal repair in epithelium and stromal layers in stem cell treated eyes, which showed more epithelial layers and enhanced wound healing performance of Ki67, E-Cadherin, and α-SMA. Our study shows the potential clinical and histological advantages in the topical ADSC treatment for corneal lesions in mice.

## 1. Introduction

The cornea provides a protective barrier and clear optical pathway for the visual system. This avascular and transparent structure is composed of three layers, which include the outer most non-keratinized stratified epithelium, stroma, and single-layered endothelium. Most corneal diseases and trauma involve the epithelium and stroma and include immune disorders, chronic inflammation, infection, iatrogenic procedures (i.e., laser refractive surgery), etc. These ocular disorders tend to cause severe inflammation, persistent epithelium defects, neovascularization, conjunctivalization, persistent corneal opacities, and scarring, which can all lead to permanent vision loss [1,2,3]. 

The most common intervention for restoring corneal clarity and function is full thickness penetrating or lamellar keratoplasty. However, there are great limitations related to little availability of donor tissue and costs and complications related to surgery (i.e., graft failure, chronic inflammation, numerous follow-up, etc.). Alternative surgical techniques include amniotic membrane transplantation and conjunctival limbal autograft from the healthy fellow eye, but surgical outcomes and limitations are similar to keratoplasty [1]. Studies have shown that autologous serum [4] autologous plasma rich in growth factors [5,6] and other novel topical treatments [7,8] can be beneficial in patients with various ocular surface disorders due to inflammation, severe dryness, and persistent epithelial defects [4,5,6]. However, long-term clinical efficacy multicenter data and standardized preparation protocols are lacking. Moreover, therapeutic effects tend to be superficial, limiting and symptomatic involving the corneal epithelial and not directed on lesion induced stromal scarring and damage. 

In the past decade, stem cell therapy has been proposed in almost every branch of medicine, ranging from cardiology, neurology, plastic surgery, dentistry, etc. [9,10,11,12] Similar to bone marrow derived stem cells, numerous studies have shown that adipose derived stem cells (ADSC) are pluripotent and have the capability of differentiating into multiple mesodermal cell lineages expressing specific markers and proteins [12,13,14]. The interest in ADSC has greatly increased considering that it is easy to obtain large quantities of autologous tissue in a rapid minimally invasive liposuction procedure that requires only local anesthesia. Moreover, ADSC are extremely more abundant in adipose tissue than MSC in bone marrow [15]. 

Numerous studies based on ADSC obtained from processed human lipoaspirate to treat corneal lesions in various animal models have shown stem cells to be able to differentiate and produce corneal specific proteins, thus enhancing wound healing and maintaining transparency [16], while providing inhibition of inflammation [3] and angiogenesis [2]. ADSC can be placed in corneal stromal pockets. However, surgical manipulation of cells and of the host cornea can be potentially damaging and may not be of potential clinical use in a clinical setting. 

Topical stem cell therapy may be of potential clinical use considering that ADSC are abundant, cost efficient, multipotent, non-invasive, and beneficial in various phases of wound healing including cellular remodeling, changes to tear composition, cell migration, proliferation, epithelial reattachment, and stromal remodeling. Our previous study reported the topical use of ADSC in chemically induced corneal lesions in rats [17]. These preliminary results showed that stem cell treated eyes had significantly smaller epithelial defects at each time point, with better and faster re-epithelization and less inflammatory response compared to other treatment arms. The ADSC group seemed to show improved corneal wound healing. However, a small number of animals with chemically induced lesions in a brief time were considered in the treatment and in the clinical assessment to provide a semi-quantitative assessment of data. The purpose of our current study was to assess a greater number of eyes over a longer time period using a reproducible laser-induced corneal wound in mice in addition to provide a comparison of epithelial repair, stromal haze, inflammation, and quantitative histological analysis between treatment groups.

## 2. Material and Methods

### 2.1. Animals

Forty black male mice C57BL/6 (30–40 g) purchased from Charles River Laboratories (Barcelona, Spain) were used in the experiments. Animals were housed with a 12-h light–dark cycle with ad libitum access to food and water. Animal care and experiments were carried out in accordance with the guidelines of the Spanish Ministry of Health for Animal Care (RD 53/2013) and European Commissions (2003/65/EC). 

Prior to induction of lesion and treatments, mice were anesthetized by intraperitoneal injection of 80 mg/kg ketamine hydrochloride (Imalgène 500, Merial, Lyon, France) and 5 mg/kg xylazine hydrochloride (Rompun, Bayer HealthCare, Kiel, Germany). Topical anesthesia was induced by 0.4% oxibuprocain eye drops (Novesina, Novartis, Varese, Italy). Animals were euthanized with an overdose of sodium pentobarbital (Dolethal, Vétoquinol, Lure, France) and verified by cervical dislocation. 

### 2.2. Isolation and Preparation of Adipose Derived Stem Cells

Human subcutaneous abdominal adipose tissue was obtained from healthy patients (aged 35–64 years) undergoing elective lipoaspiration surgery with informed oral and written consent under a protocol approved by the Institutional Review Board (IRB) of the University of Udine, in accordance with the guidelines of the Tenets of the Declaration of Helsinki. Patients were screened and resulted negative for HIV, hepatitis B and C virus, and syphilis.

Isolation, preparation, flow cytometry, immunofluorescence, and multilineage differentiation of ADSC have been reported in our previous studies [12,17,18].

For the in vivo experiment, we employed ADSC obtained from a 35 year old female patient, at the third passage in culture, in their undifferentiated state.

### 2.3. Blood Serum

Human serum was prepared in accordance to the Azienda Ospedaliero Universitaria Santa Maria della Misericordia protocol [17]. In brief, 500 mL of whole blood from one healthy young male donor (45 years old) was collected into sterile 9 mL tubes after written informed consent. The patient was screened and resulted negative for HIV, hepatitis B and C virus, and syphilis. The containers were left standing in an upright position to ensure clotting for at least 30 min at room temperature, then centrifuged at 2000× g for 10 min. The supernatant serum was removed under sterile conditions in a laminar flow hood with sterile disposable syringes. The vials were frozen and left to thaw for 24 h before treatment. In accordance to our hospital protocols on clinical treatment for severe dry eye syndrome, we used undiluted whole serum. 

### 2.4. Laser Induced Corneal Wound

After intraperitoneal and topical anesthesia, corneal lesions were performed on both eyes of 40 mice by laser induced photorefractive keratectomy (PRK) [19]. To induce the epithelial and stromal laser lesion, each eye was placed under a Wavelight Allegretto Wave PR-020407 excimer laser (Wavelight GmbH, Alcon, Erlangen, Germany). The PRK ablation parameters included a diameter of 2 mm to the central optic zone and a total depth of 45 µm to induce a uniform lesion affecting both epithelium and stroma. The laser began nasally and progressed temporally. 

### 2.5. Treatment Regimen 

All animals were treated with topical eye drops of azythromycin 1.5% (Azyter, Laboratoires Thea, Clemrmont-Ferrand, France) for antimicrobial prophylaxis two times daily for three days after lesion [19]. Eighty eyes of 40 mice were divided in four treatment groups (*n* = 20 eyes per group), which included control, stem cells, basic serum, and plasma rich in growth factor (PGRF). Data from the PGRF (not shown) were intended and collected for a different ongoing study. Control eyes received only antibiotic eye drops. The other 3 groups also received topical treatment applied three times a day for five consecutive days. Topical drops were administered with a delay of at least 5 min between applications for multiple treatment regimens. Stem cell topical eye drops were prepared daily with 1 × 10^5^ cells suspended in 25 µL HBSS/treatment [16]. The basic serum group received topical application of 25 µL of 100% human serum. 

### 2.6. Ocular Surface Evaluation 

Upon topical anesthesia, each treated eye was examined with a stereo biomicroscope before application of topical treatment at 30, 54, 78, 100, and 172 h after lesion (also referred to in this study as day 1, 2, 3, 4, and 7, respectively). This was done at each time point to assess corneal inflammation, opacities, and other anterior surface complications (i.e., infection, perforation, etc.). Fluorescein sodium solution (Colircusí Fluoresceína, Alcon Cusí, Barcelona, Spain) was used to evaluate the degree of the corneal epithelial defect. Each animal’s anterior segment was photographed with a Leica S6D stereo microscope (6.3:1 zoom and 15.0× magnification) equipped with a Leica EC3 digital camera (Leica Microsystems, Wetzlar, Germany) with and without fluorescein at each clinical assessment. The defect area was determined by the fluorescein positive remaining area under blue light (1 mm = 240 pixels) using ImageJ 1.45a software (National Institutes of Health, Bethesda, MD, USA). Based on anterior segment visualization of pupil, iris and the presence of corneal vessels with stereo biomicroscopy at each examination time point. The analysis was performed independently by two masked graders. 

### 2.7. Histological Examination

Eyes were fixed in Somogyi’s fixative without glutaraldehyde, rapidly frozen in liquid nitrogen, and preserved in OCT compound. The specimens were cut into 5 µm-thick tissue sections with a cryostat and subjected to immunofluorescence techniques. Sections were examined under fluorescence microscopy. With regards to the 33 mice of the 40 animals (66 eyes) included in the analysis, 32 eyes of 16 animals were enucleated after 78 h (day 3), while the remaining 34 eyes of 17 mice were enucleated at the end of the study at 172 h (day 7) post-lesion. The details regarding the histological preparation and assessment are reported in the Appendix section.

For immunofluorescence analysis we used antibodies to Ki67 (proliferation; 1:500; Abcam, Cambridge, UK), α-SMA (myofibroblast transformation; 1:200; Abcam), E-cadherin (assembly of epithelial cells; 1:200; Santa Cruz Biotechnology, Santa Cruz, CA, USA). Further details regarding the antibodies used in our study have been reported in the Appendix section under the heading “Histological examination” on page 14, lines 532–575.

## 3. Statistical Analysis

Normality of the data distribution was assessed with the Kolmogorov-Smirnov test. Data were expressed as median ± standard deviation. Differences of the data amongst groups were analyzed with SPSS 20.0 (SPSS Inc., Chicago, IL, USA) for Windows program using Kruskal-Wallis and Friedman test. Multiple comparisons were performed with Dunnett’s test. A *p* value of < 0.05 was considered to be statistically significant.

## 4. Results

### 4.1. Clinical Outcomes

The PRK lesion was performed uneventfully in all mice eyes. Immediately after the laser ablation, the treated areas showed a whitish uniform, hazy appearance. There were no signs of neovascularization or perforation in all eyes. Three animals (#24, 29 & 39) died of hypothermia at 30 h after anesthesia, and four animals (#6, 12, 38 & 28) were found dead in the cages (probably due to natural causes) before the endpoint on the 7th day. A total of 33 of 40 animals (66 eyes) were considered in the statistical analysis: 18 stem cell treated eyes, 15 basic serum eyes, and 17 control eyes. Of these, 16 (32 eyes) underwent treatment for three days and sacrificed at 78 h, and the remaining 17 animals (34 eyes) completed the five-day treatment regimen and then sacrificed at 172 h. 

**Figure 1:**
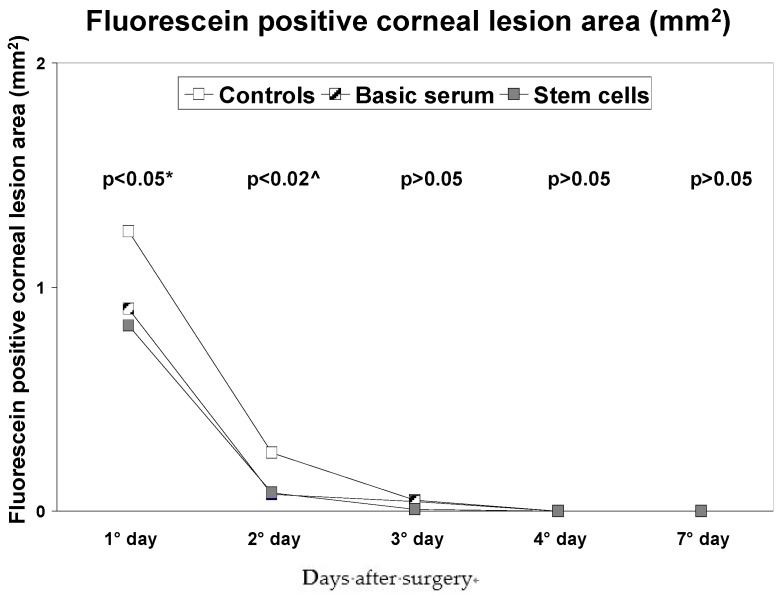
Intragroup comparisons of median epithelium defect area over time for each treatment group in the mice eyes. * = statistically significant difference between stem cells and control groups; ^ = statistically significant difference between controls and the other groups.

**Figure 2:**
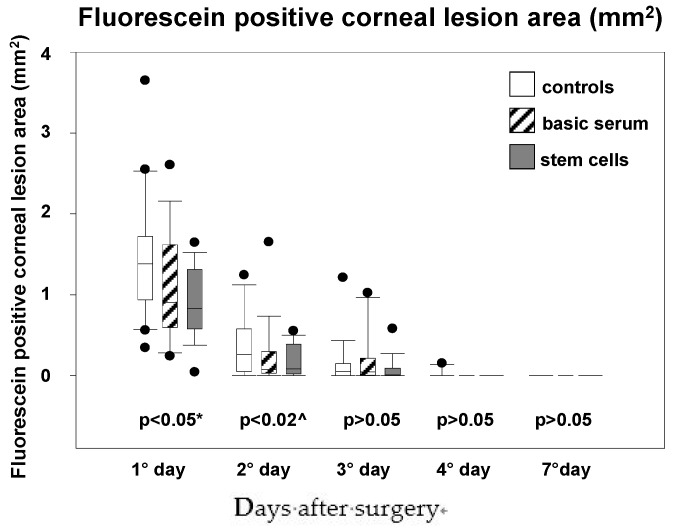
Box plots of median epithelium defect area over time at each time point for each treatment group in the mice eyes. * = statistically significant difference between stem cells and control groups; ^ = statistically significant difference between controls and the other groups.

Partial re-epithelization was seen in all mice eyes at the first time point at 30 h. All eyes were completely re-epithelized by 100 h (Table 1a & Figure 1). On the first day, the fluorescein positive corneal lesion area was significantly smaller in the stem cells groups than the control eyes (Table 1a & Figure 1 & Figure 2; *p* < 0.05); on the second day, it was significantly larger in the controls, yet comparable between stem cell and serum treatment groups (Table 1a & Figure 1 & Figure 2; *p* < 0.02). No differences were found amongst groups on the other days. 

**Figure 3:**
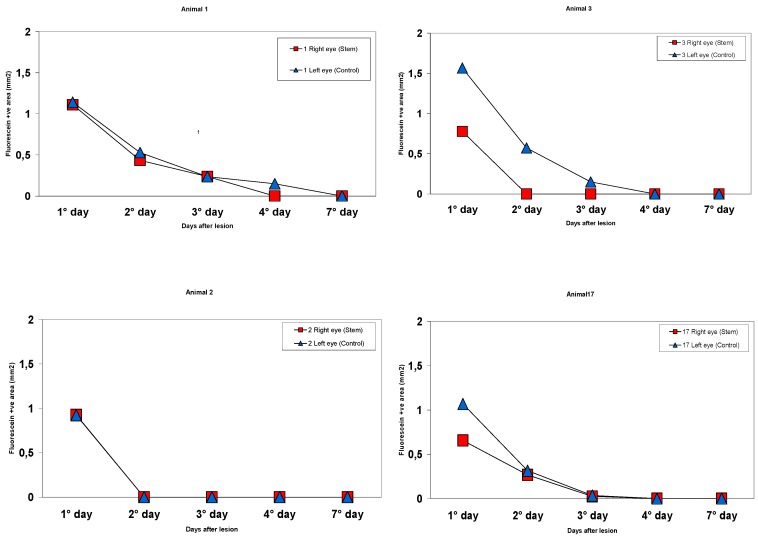
Fluorescein positive areas at each time for animals # 1, 2, 3, and 17. For intraindividual comparisons, the right eye (red squares) of each mouse was treated with stem cells and the left eye (blue triangles) was treated with the control regimen. The stem cell eyes tended to show better epithelial wound healing than the control eyes in the first three days with smaller fluorescein areas and faster total re-epithelization.

Inter-individual differences were seen in the mice, even amongst eyes with the same treatment. To limit this variability, several mice were treated with stem cells on the right eye and control on the left. The stem cell treated eyes tended to show faster wound healing with smaller defect areas at most of the earlier time points in all these eyes. Figure 3 shows examples of the fluorescein positive areas at each time point for these eyes. 

Qualitative histology assessments were performed in 27 eyes of 15 mice eyes at 78 h (day 3) after lesion (10 control, 8 stem, 9 basic serum) and 28 eyes of 15 mice eyes at 172 h (day 7) after lesion (8 control, 10 stem, 10 basic serum). 

### 4.2. Histological Outcomes 

**Figure 4:**
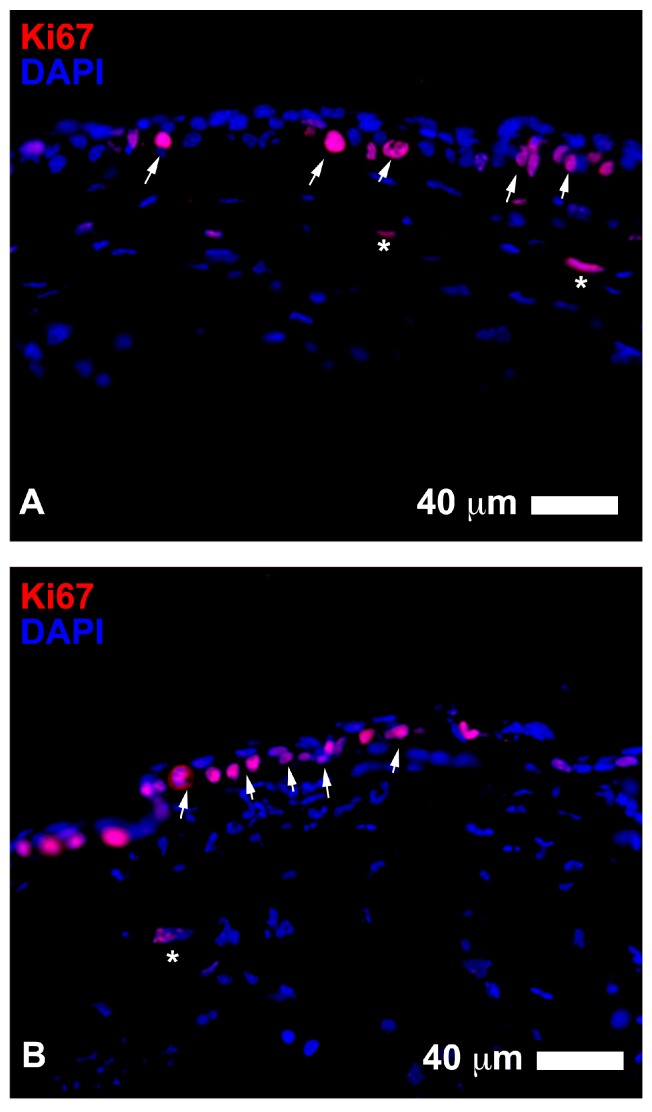
Images of Ki67 positive cells (in red) in the central epithelium of wound healing corneas treated with adipose-derived stem cells ADSC for three days (**A**) compared to the control eyes (**B**). Both groups showed a high number of proliferating cells in the basal cell layer of the regenerating epithelium (arrows). Note a slightly higher level of organization in stem cell treated cornea. Some proliferating cells could be found in the stroma (asterisks) (Scale bars: 40 µm).

**Figure 5:**
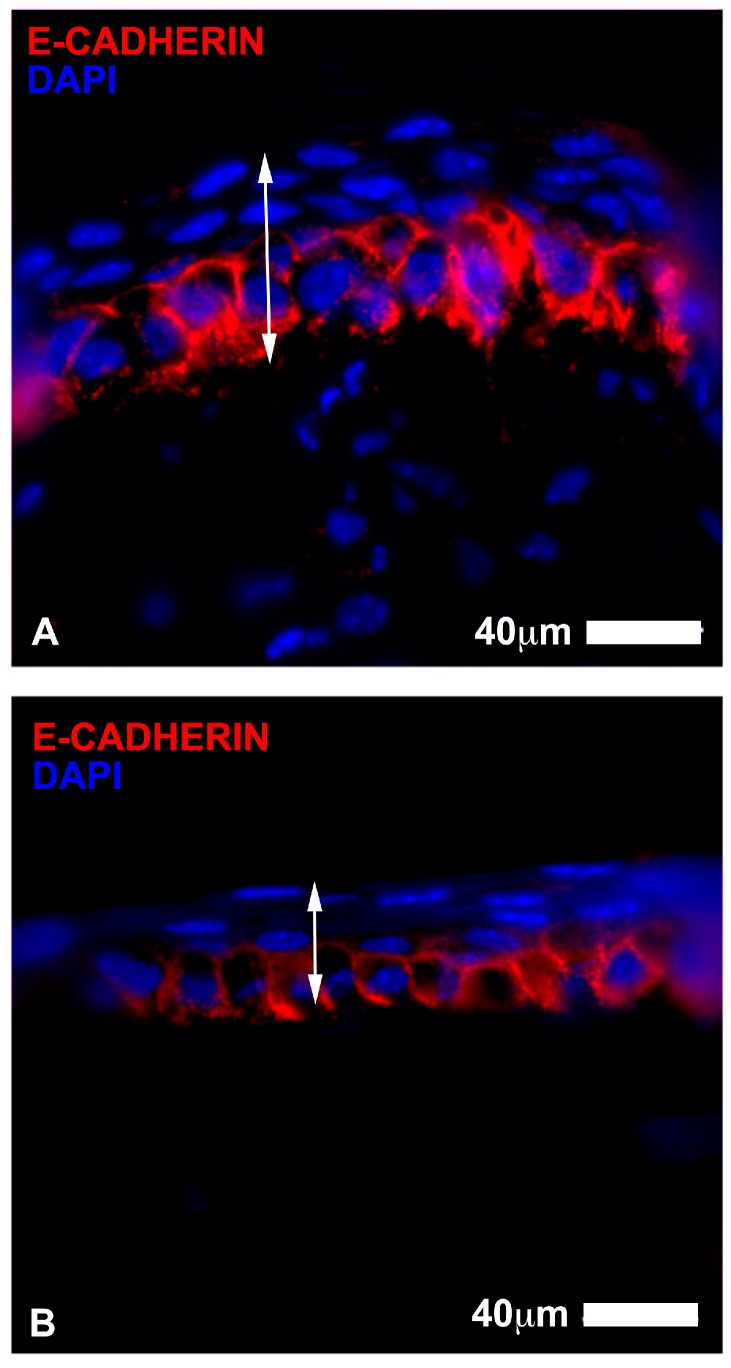
Corneal epithelium wound healing after three days assessed with E-Cadherin immunostaining. (**A**) represents an eye treated with ADSC. E-Cadherin (marked in red) appeared to be distributed in the basal cell layer of the epithelium and accumulated in the cytoplasm. Elongated irregular processes could be seen in the stroma; some cells presented E Cadherin staining; (**B**) represents a control eye, which showed a similar pattern of distribution of E Cadherin staining in the cornea. Nuclei are counterstained with DAPI (in blue). Note the difference in the number of layers between stem cell treated group and untreated corneas (Scale bar 40 µm).

With regards to the epithelium histological assessment after three days, the stem cell group showed a slightly increased number of epithelial cell layers (Figure 4 and Figure 5). There were three layers of epithelial cells in the peripheral region of the damaged cornea, while about four layers of cells in the central post-injured area. The number of Ki67 cells in the peripheral epithelium was similar amongst groups, however, the stem cells treated eyes showed less of these actively replicating cells in the epithelium, yet more in the stroma underlying the lesion when compared with the other groups (Table 1b). E-Cadherin protein accumulated in the basal cell layer of the epithelium and was present in the cytoplasm (Figure 5). This localization suggests that the epithelium was not stabilized and was still involved in a reorganization process [20,21,22]. In the control eyes, there were only three layers of epithelial cells and a slightly less number of Ki67+ cells in the peripheral epithelium (Table 1b). The E-Cadherin pattern was similar to that described in the stem cell treated eyes (Figure 5). In the basic serum eyes, there were 5 to 6 layers of epithelial cells and a higher number of Ki67+ cells across the extension of the epithelium. The E-Cadherin pattern was diffuse in the basal cells of the epithelium, similar to the stain observed in the other groups.

**Figure 6:**
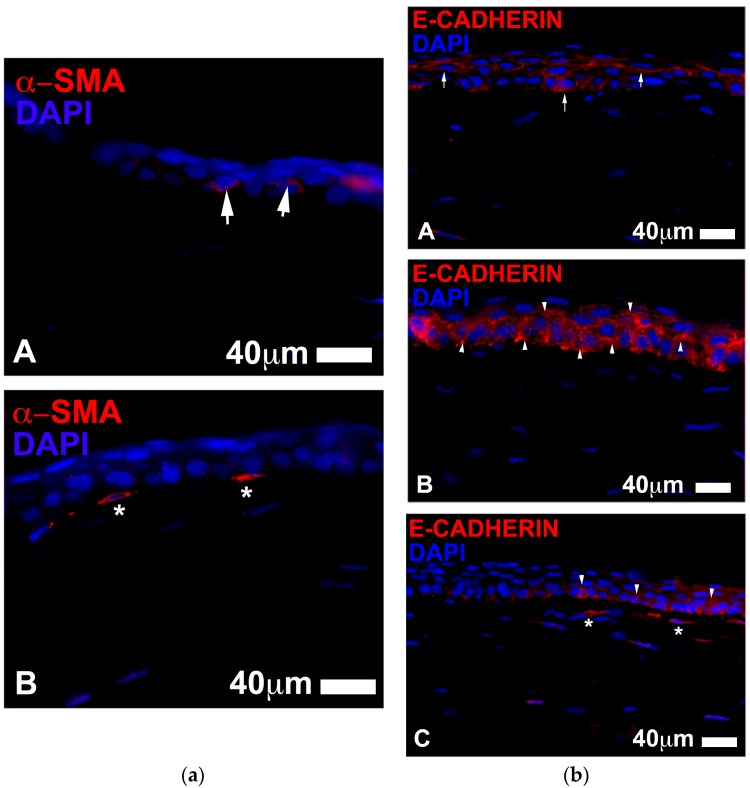
(**a**) ADSC treatment (**A**) seemed to retard the onset of myofibroblast appearance when compared to the controls eyes (**B**) during the initial stages of the wound healing process (three days after injury). There tended to be scarce or no myofibroblasts in the stroma in the ADSC treated eyes; only a diffuse staining for α-SMA (in red arrows) was visible in the basal cells of the central epithelium (**A**). In contrast, several α-SMA^+^ myofibroblasts (shown as elongated cells stained in red asterisks) were present in the upper stroma of the control eyes (Scale bars: 40 µm). (**b**) E-Cadherin staining (in red) 7 days after injury. E-Cadherin appeared to be located bordering the epithelial cells and rarely accumulated in the cytoplasm in the ADSC treated eyes (**A**). The control corneas showed a different staining pattern for E Cadherin, which accumulated in the cytoplasm of the epithelial cells (**B**). A similar location of epithelial E-Cadherin was found in the basic serum treated eyes, which tended to accumulate in the cytoplasm of the basal cells (**C**). There also appeared to be more E-Cadherin^+^ cells in the stroma of the basic serum treated eyes. (Scale bar 40 µm).

With regards to the stromal histology at three days, all eyes showed E-Cadherin-positive cells indicating the presence of migratory elements in the stroma, probably involved in reparative processes. Moreover, a small number of myofibroblasts (α-SMA positive profiles) were found in the stromal under the epithelium in the lesion area. These myofibroblasts were more frequent in the control corneas when compared to those found in the basic serum treated eyes. These myofibroblasts were scarce and not frequently seen in the stem cell treated eyes (Figure 6a).

**Figure 7:**
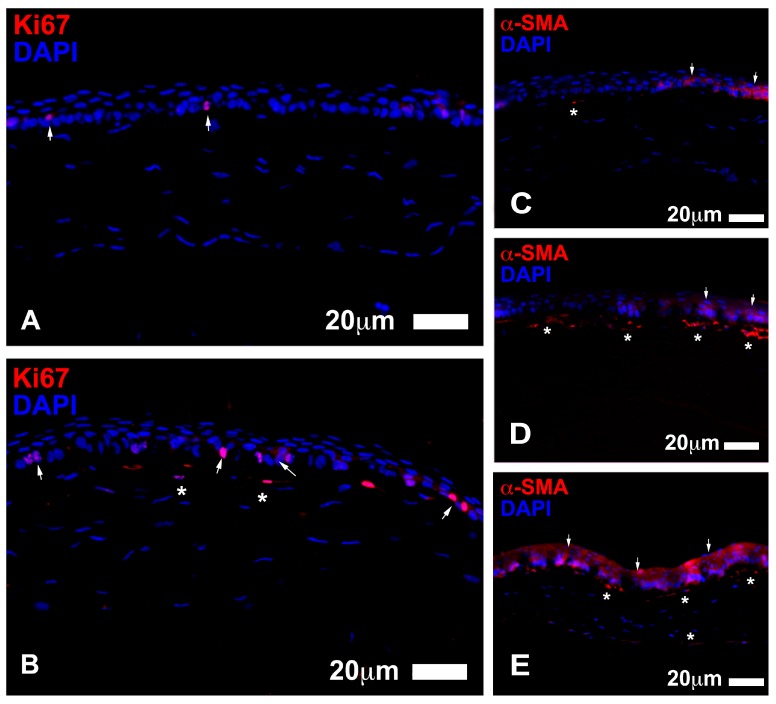
Left panel shows examples of proliferative events in the central epithelium 7 days after injury. In the ADSC treated eyes (**A**), there were scarce residual amounts of Ki67+ cells (in red, arrows), which can normally found in the epithelium uninjured corneas. There were no proliferative elements found in the stroma. In contrast, the control eyes (**B**) showed numerous Ki67 positive cells located both in the central epithelium (arrows) and stroma (asterisks). Right panel shows α-SMA staining (in red) during corneal wound healing 7 days after injury. The ADSC eyes showed a very low number or absence of myofibroblasts in the stroma ((**C**), asterisk). Control eyes exhibited strong staining to α-SMA in the upper stroma with numerous filiform myofibroblasts ((**D**), asterisks). The basic serum treated eyes also showed a high number of α-SMA+ myofibroblasts in the stroma ((**E**), asterisks). The epithelium showed a diffuse staining to α-SMA in all cases (arrows), which varied and did not appear to be influenced by treatment (Scale bars: 20 µm).

After seven days of lesion, the stem cell treated eyes showed epithelium that was similar to uninjured epithelium, composed of 4 to 5 layers of uniform and perfectly structured epithelial cells (Figure 6b). E-Cadherin protein was localized at the periphery of epithelial cells, similar to what is normally found in healthy uninjured epithelium, thus indicative of a stabilization of the corneal regenerating epithelium [22]. The number and distribution of proliferating Ki67 positive cells were similar to that measured in normal mouse corneal epithelium. The control eyes showed the same structure of epithelium; however, there appeared to be at least one additional layer and more Ki67 positive cells (Table 1b, Figure 7). The basic serum treated eyes showed about 5 to 6 layers in the epithelium and a higher number of Ki67 positive cells (Table 1b).

The stromal histology after seven days (Figure 7) showed that the stem cell treated had little or no E-Cadherin positive elements in the stroma. The E-Cadherin positive elements, however, appeared to be abundant in the basic serum treated eyes and control eyes, which can be indicative of migratory events in the stroma. After seven days, there was a significant reduction in the quantity of α-SMA^+^ myofibroblasts in stem cell treated group (4.09 ± 0.48 cells) compared to control (10.85 ± 0.76 cells; *p* < 0.001) and, to a lesser extent, compared to basic serum group (6.27 ± 0.65 cells; *p* < 0.05).

## 5. Discussion

Similar to our previous studies, ADSC were obtained from human adipose tissue aspirates following a protocol optimized for the isolation and in vitro expansion of human multipotent adult stem cells [12,17]. ADSC expressed the pluripotent state-specific transcription factors Oct-4, Nanog and Sox 2 [12,17]. ADSC highly expressed CD90, CD105, CD73, however, were mainly negative for the hematopoietic markers CD34 and CD45. These cells displayed multipotency. Further details regarding ADSC are reported in the Appendix section. Our results show that ADSC seem to enhance corneal wound healing induced by laser ablation when compared to traditional topical therapy. 

In accordance to our previous study based on chemical induced corneal lesions in rats [17], the percent of epithelium fluorescein positive damage in eyes treated with stem cells (with or without serum) was smaller at each time point, which was statistically significant when compared to the control mice eyes (Table 1a). Stem cell treated eyes reached complete epithelium closure faster than the control eyes (Figure 1). The results showed statistically smaller lesions after day 1 in the stem cell group, which was comparable to the basic serum treated eyes but less than that observed in the control group on days 2 and 3 (Table 1a, Figure 1 & Figure 2). 

With regards to the mice histological data, the presence of more layers of epithelial cells in the early stages of repair in the stem cell treated group compared to the control eyes could be related to an improvement in the re-epithelization of the corneal surface after injury. The epithelium under the lesion had a smaller number of Ki67 actively replicating cells in the stem cell eyes, yet the stroma appeared to have significantly more than the other groups (Table 1b), which may be indicative of earlier stromal repair and post-mitotic epithelium layer reformation in this group at three days. Although these epithelial layers covered the entire corneal surface, these cells appeared to be undergoing reorganization during the initial period after lesion, as deduced by the elevated rate of proliferation of basal epithelial cells in the central region of the cornea and the localization pattern of E-Cadherin protein in the epithelium [21,22]. E-Cadherin did not appear to be distributed in the periphery of epithelial cells, as expected in stable uninjured epithelium; however, it mostly accumulated in the cytoplasm of basal epithelial cells. This location of E-Cadherin suggested an ongoing reorganization process of the epithelial layers [20,21,22]. 

Although all eyes exhibited complete re-epithelization macroscopically after seven days, the histological evaluation showed that the epithelium of individuals treated with stem cell contained four or five layers, which is the number of layers normally found in uninjured mouse corneal epithelium. The number of dividing cells (Ki67+) in the central region was very low, which resembled a normal cornea with a well-established epithelium structure. The cytoarchitecture of the epithelium was that of a normal epithelium, showing cuboidal cells in the basal layer, with a few layers of low cuboidal cells positioned on top, followed by 1–2 outer sheets of flattened wing cells. E-Cadherin appeared to be located externally bordering epithelial cells and was observed in all layers. In comparison, the control eyes had at least one additional layer of epithelial cells, which may be indicative of an incomplete or altered repair process. The eyes treated with the blood derivatives PRGF (data not shown) and basic serum showed five to six layers of epithelial cells, which may be indicative of an ongoing re-epithelization and possible delay in the cessation of cell proliferation in the central region of the cornea, as seen with the presence of numerous Ki67+ cells.

The stroma of mice eyes treated with stem cells had a notably lower density of myofibroblasts (α-SMA^+^ elements) compared with control and basic serum eyes at both three and seven days, which may be related to the slightly better corneal transparency and lower haze in the stem cell eyes (based on a qualitative comparative analysis between groups, data not shown) [23,24,25,26,27]. E-Cadherin+ cells were not found in the stroma of stem cell treated eyes and controls after seven days, yet were abundant in the PRGF and serum treated eyes. The lack of E-Cadherin+ cells can be indicative of a stabilization of the stromal cytoarchitectural organization. In contrast, the stroma of the blood derived treated groups continued to have reorganizations events and mobility of cellular elements in the stroma. The physiological roles of ADSC are diverse and promising in tissue regeneration and wound healing [13,14,28]. ADSC obtained from lipoaspirate have been shown to meet the minimal set of 4 criteria proposed by the Mesenchymal and Tissue Stem Cell Committee of the International Society for Cellular therapy [29] used to define functional human MSC [12,13,18,30] In addition to their extensive proliferation potential and multilineage differentiation, ADSC can interact and affect the immune system response to injury, by the down-regulation of proinflammatory factors and production of several trophic factors [23,31,32]. Several reports have shown the immunoregulatory properties of MSC, which include the inhibition of T-cells [33], increase in tumor necrosis factor (TNF) from dentritic cells [34], increase in regulatory T-cells [34], block of antigen producing cell maturation [35], increase in immunosuppressive cytokine interleukin (IL)-10 and TNF-β and decrease in IL-2 [2]. The endocrine function of adipose tissue is evident through the secretion of numerous growth factors (GF) like epidermal GF, vascular endothelial GF, basic fibroblast GF, keratinocyte GF, and platelet-derived GF [30]. 

There are limited studies in current literature regarding the use of ADSC in ocular surface and stromal wound healing [36,37,38,39]. Arnalich-Montiel showed that human ADSC could regenerate into corneal tissue in situ when inserted in a laser induced intrastromal corneal pocket in a rabbit model [16]. The transplanted cells appeared to be safe, maintained corneal transparency and caused no immune reaction [40]. The transplanted multipotent stem cells acquired similar characteristics to keratocytes [41]. Studies have shown that topical application of stem cells are easy, and may prove to be clinically acceptable and effective in acute phases [42,43]. 

The presence of corneal limbal stem cells (LSC) was first discovered in the late 1980’s [44]. These slow-cycling subpopulation of epithelial basal cells located in the peripheral limbus of the cornea were found to have a substantial proliferating capacity. Autologous and allogenic LSC transplants are surgical option [45], however, donor tissues are limited and autologous transplants may give rise to iatrogenic damage to the healthy fellow eye or at times the fellow eye is not healthy. Recent studies have reported the presence of MSC in the human limbal biopsies having similar immunophenotype and immunocytochemical markers to those present in bone marrow derived MSC [46]. 

With regards to the clinical use of ADSC for ocular surface wounds in humans, only a single case report has been published by a group in Greece [47]. A young male underwent an experimental treatment involving topical application of autologous ADSC, which was obtained by lipoaspiration of subcutaneous adipose tissue from the lumbar area. The ADSC were isolated from the lipoaspirate and applied to the bottom of the ulcer, followed by closure of the lid with a pressure eye patch for 24 h. Corneal healing was observed after 11 days. At six months after treatment, the patient still did not require surgery, visual acuity was improved to 20/40, central corneal thickness was 476 µm, and corneal transparency improved with mild residual anterior stromal opacification. 

## 6. Conclusions

Our mice experiments based on a laser-induced lesion confirmed that stem cell treated eyes had significantly smaller epithelial defects at each time point, with better and faster re-epithelization and less inflammatory response compared to other treatment arms, which was already reported in our preliminary rat study based on a chemical induced lesion model. Literature in this field is limiting, thus additional studies using a different animal model and different type of lesion was proposed in this study to confirm preliminary experiments and report additional data. Our mice experiments were based on a larger group of animals assessed for a longer follow-up compared to our preliminary rat studies. Our study adds to the limiting literature currently available in this field by confirming the biosafety, immunogenicity, and potential clinical and histological advantages in using this mode of stem cell treatment. Future studies involve the assessment of epithelial recovery, inflammation, corneal haze, and quantitative histological assessments compared in different treatment arms. Although the exact mechanisms are not known, these cells are multipotent and have the potential to differentiate toward a keratocyte stromal lineage and therefore theoretically appear to be a promising therapeutic alternative and advantageous compared to treatments currently utilized in clinical practice. Most studies have shown ADSC to be promising in animal models, which can serve as a great stepping stone in addressing the topical therapeutic use of stem cells in humans.

## Figures and Tables

**Table:** (**a**)

Time after Lesion	Controls	Basic Serum	Stem Cells	*p*
Day (hours)	(Median ± SE)	(Median ± SE)	(Median ± SE)	Value *
Day 1 (30 h)	1.25 ± 0.80 ^a^	0.90 ± 0.68	0.83 ± 0.42	0.048
Day 2 (54 h)	0.26 ± 0.39 ^b^	0.07 ± 0.40	0.08 ± 0.20	0.018
Day 3 (78 h)	0.05 ± 0.29	0.04 ± 0.32	0.01 ± 0.14	0.127
Day 4 (100 h)	0.00 ± 0.05	0.00 ± 0.00	0.00 ± 0.03	0.202
Day 7 (172 h)	0.00 ± 0.00	0.00 ± 0.00	0.00 ± 0.00	1.000
*p* value ^	0.0001	0.0001	0.0001	

* = Kruskal-Wallis test; ^ = Friedman test; ^a^ = significantly higher than stem cells group; ^b^ = significantly higher than the other groups.

**Table:** (**b**)

Group	Number of Ki67 Cells ± Standard Deviation		
	Peripheral Epithelium	Central Post-Lesion Epithelium	Central Stroma
**Three Days after Lesion**			
Control (*n* = 10)	112.25 ± 17.00 ^a^	69.00 ± 8.00	7.00 ± 2.10
Stem (*n* = 8)	120.00 ± 8.90 ^a^	37.30 ± 6.33 ^b^	18.00 ± 4.43 ^c^
Basic serum (*n* = 9)	131.40 ± 9.77 ^a^	72.30 ± 7.60	9.55 ± 2.89
*p* value ^	0.027	0.0001	0.001
**Seven Days after Lesion**			
Control (*n* = 8)	67.00 ± 7.89	24.00 ± 5.40 ^a^	4.50 ± 1.66
Stem (*n* = 10)	61.00 ± 5.22 ^c^	16.50 ± 2.88 ^a^	1.33 ± 0.54 ^b^
Basic serum (*n* = 10)	65.50 ± 5.40	27.60 ± 5.74 ^a^	4.30 ± 1.25
*p* value ^	0.022	0.001	0.001

^ = Kruskal-Wallis test; ^a^ = significantly different than the other groups; ^b^ = significantly lower than the other groups; ^c^ = significantly higher than the other groups.

## Appendix A

### Appendix A.1. Animals

Our decision to use both eyes was not solely based on a cost issue but based on a similar experimental model used in our previous study published in Current Eye Research [17]. This was done in accordance to the 3R statement of the European Community (reduce, replace, refine) that encourages the use of fewer experimental animals, and to compare treatments on the same animals to reduce inter-animal variability, thus both eyes were also used in the experiment. In addition, we obtained the approval of the Animal Ethical Committee of the University of Oviedo (Spain).

### Appendix A.2. Histological Examination

Eyes were fixed in Somogyi’s fixative without glutaraldehyde for 3 h, cryoprotected overnight in 30% sucrose solution, embedded in OCT compound and frozen in liquid Nitrogen. The entire frozen eyeball was placed on a cryostat chunk and oriented to obtain transversal sections of the cornea. Cryostat sections with a thickness of 5 µm were collected on Superfrost microscope slides (Thermo Scientific, Rochester, NY, USA) and stored frozen.

For each eye, three selected histological sections in the central region of the injured cornea were chosen to demonstrate each marker used to evaluate the quality of corneal repair, which included:

1. Polyclonal antibody against Ki67 produced in rabbit (Abcam, Cambridge, UK): This antibody is present in the nuclei of actively dividing cells and can be used as a measure of the number of proliferating cells in a repairing process [48].
2. Polyclonal antibody against α Smooth Muscle Actin (α-SMA) produced in rabbit (Abcam, Cambridge, UK): The α-SMA presenting cells are identified as myofibroblast during the wound healing process [24,25,49].
3. Polyclonal antibody against E-Cadherin produced in rabbit (Santa Cruz Biotechnology, Santa Cruz, CA, USA): The E-Cadherin protein locates normally bordering epithelial cells, thus allowing a visualization of the cytoarchitecture of a multilayered epithelium [20,21].

Fluorescent immunohistochemical techniques were used to assess these markers. Selected cryosections were carefully thawed in a water bath; slides were placed in a humidity chamber and washed three times in PBS (Phosphate Buffered Saline) with Triton X-100 (0.03%). A brief incubation with a blocking medium (1% bovine serum albumin and 10% normal goat serum; Vector Laboratories, Burlingame, CA, USA) was added to avoid nonspecific binding of the secondary antibody. The blocking medium was then removed by washing the slides three times with PBS with Triton X-100 (0.03%). The sections were incubated overnight at 4 °C with the appropriate primary antibody at a specific dilution (Anti-Ki67 at 1:500; Anti-αSMA at 1:200; and, Anti-E-Cadherin at 1:100). Sections were then washed again three times in PBS and incubated with a fluorescent secondary antibody (anti-rabbit Alexa Fluor 594 raised in goat, Molecular Probes, Grand Island, NY, USA) in a dilution of 1:500 for 2 h at room temperature in the dark. Three additional washes were then used to eliminate the excess of fluorescent antibody. Nuclei were counterstained with 2 µg/mL 4′,6-diamidino-2-phenylindole dihydrochloride (DAPI; Molecular Probes, Grand Island, NY, USA) for 10 min and slides were mounted with Dako fluorescence mounting medium (Dako, Glostrup, Denmark) and coverslips. Slides were then identified and encoded with a numerical system and stored at 4 °C.

The analysis of the wound healing parameters was performed on histological images using a Leica DM6000 microscope equipped with epifluorescence using a 20× objective (numerical aperture 0.45) and AF6000 software (Leica Microsystems, Wetzlar, Germany). Measurements were performed in a semiautomated manner using ImageJ 1.45a software (National Institutes of Health, Bethesda, MD, USA). Two independent masked observers performed the analysis. Data were collected and decoded, and statistical analysis was performed using InStat 3.1a software (GraphPad Software Inc., La Jolla, CA, USA).

### Appendix A.3. Adipose-Derived Stem Cells (ADSC)

Human subcutaneous abdominal adipose tissue was obtained from thee healthy female patients (aged 35, 52, and 64 years) undergoing elective lipoaspiration surgery. ADSC used in the treatment group, however, came from a single healthy donor (youngest female aged 35 years) and were employed, at the third passage in culture, in their undifferentiated state. Cells were characterized in accordance to the study reported by Domenis et al. in 2015 [29]. Specifically, cells satisfied the minimal criteria of ISCT. Moreover, they were checked for additional markers, such as the expression of pluripotent state-specific transcription factors. Although we are aware that a certain degree of controversy surrounds this latter feature, Chih-Chien Tsai et al. [50] has shown that Oct-4 is not only expressed but also required for MSC self-renewal. Additionally, other independent groups showed the expression of these markers in MSC [51,52].
